# Coexpression of Matrix Metalloproteinase-7 and Tissue Inhibitor of Metalloproteinase-1 as a Prognostic Biomarker in Gastric Cancer

**DOI:** 10.1155/2020/8831466

**Published:** 2020-09-14

**Authors:** Yangyu Zhang, Lili Qin, Xiaobo Ma, Yueqi Wang, Yanhua Wu, Jing Jiang

**Affiliations:** ^1^Division of Clinical Research, First Hospital of Jilin University, Changchun, Jilin Province, China; ^2^Department of Pathology, First Hospital of Jilin University, Changchun, Jilin Province, China

## Abstract

**Background:**

Degradation of the extracellular matrix (ECM), an essential step in tumour invasion and metastasis, is mainly dependent on the activities of both matrix metalloproteinases (MMPs) and tissue inhibitors of metalloproteinases (TIMPs). This study aimed to explore whether expression of MMP-7 and TIMP-1 alone and in combination can be used as a prognostic marker for gastric cancer (GC).

**Method:**

A total of 285 patients who had undergone tumourectomy for GC were included. Gastric tumour tissues were stained immunohistochemically to evaluate expression of MMP-7 and TIMP-1.

**Results:**

Expression of MMP-7 was associated with tumour N stage and neural invasion. Multivariate Cox regression analysis suggested that expression of MMP-7 or TIMP-1 alone cannot serve as an indicator of patient prognosis; however, coexpression of MMP-7 and TIMP-1 was found to be an independent predictive factor of overall survival in patients with GC (HR = 1.74, 95% CI: 1.08-2.80). The results of stratified analysis also showed that the predictive value of MMP-7 and TIMP-1 coexpression was stronger in patients with N3 stage disease and not receiving chemotherapy.

**Conclusions:**

In conclusion, coexpression of MMP-7 and its inhibitor TIMP-1 in gastric tumour tissues is a potential prognostic marker for GC. Greater knowledge of protein expression will lead to new paradigms and possible improvements in therapeutics.

## 1. Introduction

Gastric cancer (GC) is the fifth most common malignant tumour and third leading cause of cancer death worldwide [1]. With the significant increase in discoveries and improvements in the diagnosis and treatment of GC, the mortality rate has declined notably during the past few decades. However, the prognosis of GC patients remains poor in China, with a 5-year survival rate of 35.9% [2]. Different recurrence and survival rates observed for GC patients with the same tumour stage indicate that clinical stage alone cannot sufficiently predict the biological behaviour of the tumour; novel biomarkers are needed to complement clinical parameters for individual therapy and improve survival outcomes [3].

Invasion and metastasis are estimated to account for more than 90% of patient mortality associated with solid tumours [4], and proteolytic degradation of the extracellular matrix (ECM) is an essential step in tumour invasion and metastasis [5, 6]. This degradation is mainly dependent on the activities of both matrix metalloproteinases (MMPs) and tissue inhibitors of metalloproteinases (TIMPs) [7]. MMPs are zinc-dependent proteinases that are capable of degrading almost all of the ECM in tissues and are overexpressed in many cancer tissues [8, 9]. TIMPs are secreted proteins that bind as a complex with MMPs to regulate their activity and strictly control ECM degradation [10]. However, in addition to their MMP inhibitory activity, TIMPs have various other functions, including inhibition of apoptosis by stimulating the Akt survival pathway [11], induction of angiogenesis [12] and direct stimulation of cell proliferation [13, 14], which may be directly involved in the progression and metastasis of cancer.

MMP-7 has been recognized as a critical member of the MMP family owing to its broad proteolytic activity against a variety of extracellular matrix substrates [15] and its ability to activate other MMPs (such as MMP-2 and MMP-9) [16]. TIMP-1 can block MMP-7 and coregulate ECM degradation. A number of studies have shown the poor prognostic effects of MMP-7 and TIMP-1 in gastric carcinoma [17–21], whereas others have reported conflicting results [22, 23]. Moreover, these studies focused on MMP-7 or TIMP-1 alone. Recently, Michal et al. [24] found a concomitant expression of MMP-7 and TIMP-1 in serum to be associated with worse survival in patients with colorectal cancer (CRC). However, the usefulness of MMP-7 and TIMP-1 coexpression in predicting survival outcome in GC patients has rarely been explored. Therefore, this study used immunohistochemistry to examine MMP-7 and TIMP-1 expression in gastric specimens from 285 GC patients and explore whether expression of MMP-7 and TIMP-1 alone and in combination can be used as a prognostic marker for GC.

## 2. Materials and Methods

### 2.1. Study Population

A total of 285 GC patients who had undergone tumourectomy were enrolled at the Gastric and Colorectal Department of the First Hospital of Jilin University (Changchun, China) from February 2011 to August 2018. Patients with positive surgical margins were excluded. None of the included patients had received preoperative chemotherapy or radiotherapy. The included clinical characteristics were age, gender, tumour size, postoperative chemotherapy, T stage, N stage, histological grade, vascular invasion, neural invasion, and follow-up data. Before enrolment, all participants signed informed consent forms, and this study was approved by the Ethics Committee of the First Hospital of Jilin University (Changchun, China, 2013-005).

### 2.2. Follow-Up Data Collection

Follow-up was scheduled in the third month, sixth month, and first year after the tumourectomy and then every year until death, the end of our study, or loss to follow-up. Patients who died of complications within 30 days after the operation or were lost to follow-up at the first appointment were excluded. Survival time was defined from the date of surgery to the date of death or final successful follow-up (whether the patients were alive or lost to follow-up).

### 2.3. Experimental Methods

In our study, immunohistochemistry staining was used to detect expression of MMP-7 and TIMP-1 proteins on tissue microarray slides containing formalin-fixed, paraffin-embedded tissue arrayed samples of GC patients. After routine baking and dewaxing, the slides were heated in a pressure cooker with EDTA for thirty minutes for antigen retrieval. The blocking of endogenous peroxidase was performed by using H_2_O_2_. The slides were incubated with primary antibodies against MMP-7 (ab205525, dilution: 1/150, Abcam, Cambridge, UK) and TIMP-1 (ab211926, dilution: 1/150, Abcam) for 1 hour and 30 minutes at room temperature, followed by further incubation with goat anti-rabbit IgG for 25 minutes at room temperature and washing 3 times with PBS for 5 minutes each time. 3,3′-Diaminobenzidine tetrahydrochloride (DAB) was applied to detect antigen-antibody complexes. Finally, the slides were counterstained with haematoxylin. Two pathologists who were blinded to the clinical data evaluated the immunohistochemical staining to determine expression of the two proteins. Positive expression was defined as the presence of brown granules in the cytoplasm and/or cell membrane of tumour cells.

### 2.4. Statistical Analysis

Discrete variables were compared by Pearson's *χ*^2^ or Fisher's exact test, and the results are represented as frequencies (percentages). Differences in Kaplan-Meier survival curves were assessed by the log-rank test. Hazard ratios (HRs) and 95% confidence intervals (CIs) of possible prognostic factors were assessed using the Cox proportional hazards regression model. All of the analyses were two-sided, and *P* < 0.05 was considered to be statistically significant. SPSS (version 21.0, Chicago, IL, USA) was used to conduct the analyses.

## 3. Results

### 3.1. Expression of MMP-7 and TIMP-1 and Clinicopathological Characteristics of Gastric Carcinoma Patients

**Figure 1:**
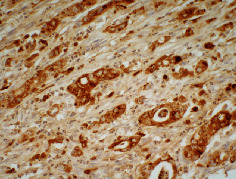
MMP-7 positive staining. The membrane or cytoplasm of gastric cancer cells was stained brown. Original magnification, ×400.

**Figure 2:**
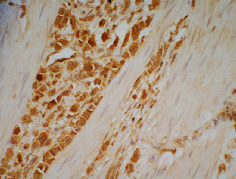
TIMP-1 positive staining. The membrane or cytoplasm of gastric cancer cells was stained brown. Original magnification, ×400.

A total of 285 patients were enrolled in the study, and the median follow-up period was 66 months. During follow-up, 168 (58.9%) patients died of GC, 106 (37.2%) patients were still alive, and 11 (3.9%) patients were lost to follow-up. There were 219 (76.8%) males in our study, and the median age of the patients was 63 (range 26-91) years. Figures 1 and 2 illustrate the positive expression of MMP-7 and TIMP-1, respectively. Among the 285 gastric tumour tissues examined, the incidence of positive MMP-7 expression was 34.4% (98/285). A total of 78 (27.4%) GC patients had positive immunohistochemical staining for TIMP-1 in tumour tissues. The results of immunostaining for the presence of MMP-7 and TIMP-1 in tumour tissues in relation to clinicopathological tumour characteristics are presented in Table 1. Expression of MMP-7 was associated with sex, with MMP-7 more frequently expressed in males than in females (*P* = 0.02). Furthermore, expression of MMP-7 was associated with tumour N stage (*P* = 0.03) and neural invasion (*P* = 0.002). MMP-7 expression increased with lymph node metastasis, and patients with tumours positive for MMP-7 often had neural invasion. The expression of TIMP-1 was not found to be associated with tumour size, histological type, depth of tumour invasion, lymph node metastasis, or lymph vascular/neural invasion.

### 3.2. Associations between Survival and Different Clinicopathological Characteristics and Expression of MMP-7 and TIMP-1

**Figure 3:**
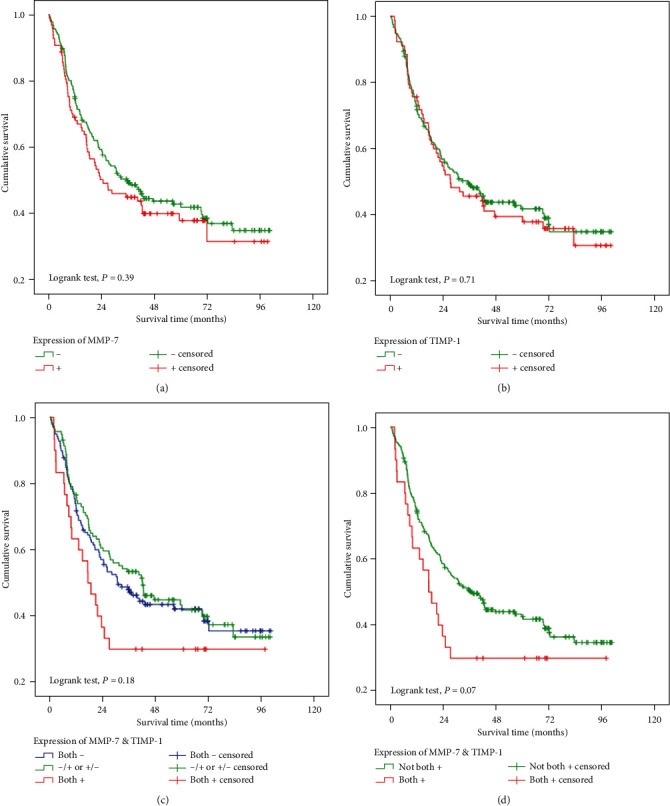
The association between expression of MMP-7 and TIMP-1 and postoperative survival in gastric carcinoma. (a) Comparison in terms of expression of MMP-7. (b) Comparison in terms of expression of TIMP-1. (c) Comparison between the three groups. (d) Comparison between the two groups. −: negative expression; +: positive expression; both −: patients whose tumour tissue expressed neither MMP-7 nor TIMP-1; −/+: patients whose tumour tissue expressed only TIMP-1; +/−: patients whose tumour tissue expressed only MMP-7; and both +: patients whose tumour tissue expressed both MMP-7 and TIMP-1.

The associations we detected between survival and different clinicopathological characteristics and different expression patterns of MMP-7 and TIMP-1 in tumour tissues of patients are summarized in Table 2. The cumulative survival time for patients with and without MMP-7 expression did not differ significantly (*P* = 0.39; Figure 3(a)). There was also no significant difference between patients with positive expression of TIMP-1 and negative expression of TIMP-1 (*P* = 0.71; Figure 3(b)). Three patterns were observed according to MMP-7 and TIMP-1 expression in gastric carcinoma tissues: A, negative expression of both MMP-7 and TIMP-1, *n* = 139 (48.8%); B, positive MMP-7 and negative TIMP-1 expression or negative MMP-7 and positive TIMP-1 expression, *n* = 116 (40.7%); and C, positive expression of both MMP-7 and TIMP-1, *n* = 30 (10.5%). The survival curves associated with the three patterns are depicted in Figure 3(c). As the curves of patterns A and B were very close and the 5-year survival rates were similar in the two groups, we combined them into one group; pattern C was treated as a separate group. The survival curves of the two groups are shown in Figure 3(d).

### 3.3. Association between Coexpression of MMP-7 and TIMP-1 and Overall Survival

A multivariate stepwise Cox regression model was performed to explore prognostic factors for GC. The results showed that age group, T stage, N stage, chemotherapy, and coexpression of MMP-7 and TIMP-1 in tumour tissues were associated with patient survival. Patients with positive expression of MMP-7 and TIMP-1 had a significantly increased risk of death compared with patients with other patterns of expression (HR = 1.74, 95% CI: 1.08-2.80) (Table 3).

### 3.4. Stratified Analysis of Expression of MMP-7 and TIMP-1 Associated with Gastric Cancer Prognosis

**Figure 4:**
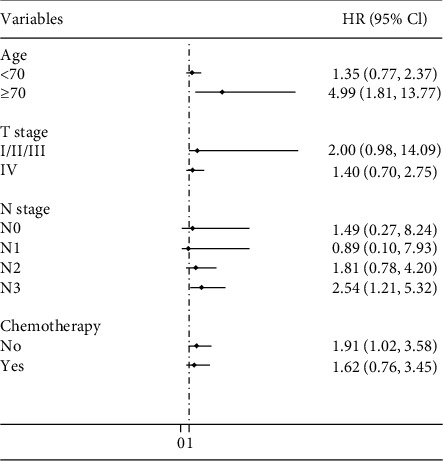
Stratified analysis of the association between coexpression of MMP-7 and TIMP-1 and gastric cancer patient survival (both + vs not both +). Age group, sex, tumour size, T stage, N stage, differentiation, histological type, chemotherapy, lymph vascular invasion, and neural invasion were used as variables in the regression model. Both +: patients whose tumour tissues expressed both MMP-7 and TIMP-1.

Associations between coexpression of MMP-7 and TIMP-1 in tumour tissues and the survival of GC patients were evaluated by a stratified analysis of age group, T stage, N stage, and chemotherapy. Compared to other patterns of expression, patients expressing both MMP-7 and TIMP-1 had a higher death risk in the subgroup of patients aged ≥70 years old (HR = 4.99, 95% CI: 1.81–13.77), patients with an advanced N stage (N stage III: HR = 2.54, 95% CI: 1.21–5.32) or patients with no postoperative chemotherapy (HR = 1.91, 95% CI: 1.02–3.58) (Figure 4).

## 4. Discussion

We analysed MMP-7 and TIMP-1 expression in GC by immunohistochemistry using 285 surgical specimens. Our results showed that although expression of MMP-7 or TIMP-1 alone may not serve as an indicator for patient prognosis, there was a significant association between concomitant positive expression of MMP-7 and TIMP-1 with poor survival of patients with gastric carcinoma. To our knowledge, this is the first study exploring the association of clinicopathological characteristics and the prognostic significance of positive expression of MMP-7 together with TIMP-1 in GC patients.

By modulating the degradation of ECM proteins and the regulation of other biomolecules in the body, MMPs are involved in many physiological and pathological conditions, such as growth, differentiation, inflammation, and angiogenesis [25, 26]. Moreover, these enzymes can affect essential steps in the biological processes of tumours and play important roles in tumour invasion and tumour spread [27, 28]. As a unique member of this family, MMP-7 exhibits a wide spectrum of substrate specificity and potential for activating a cascade of MMPs, which indicates that it is a promising biomarker in tumorigenesis and cancer progression [29–32]. Our results showed that MMP-7 was more frequently expressed in male than in female patients with GC, which may be associated with males mounting a greater and often more damaging inflammatory response to infection compared to females and with the protective effects of female sex hormones [33, 34]. Ajisaka et al. [18] found positive expression of MMP-7 in gastric tumours to be associated with deep invasion of the gastric wall, nodal metastases, and infiltration of lymph vascular invasion. Koskensalo et al. [19] confirmed that MMP-7 overexpression is associated with lymph node and distant metastases in GC. The results of our study showed that expression of MMP-7 was related to nodal metastases and neural invasion but not to depth of invasion. The role of TIMP-1 in tumour invasion and metastasis appears to be complex and, in some cases, even divergent. For example, a study conducted by Zhang et al. [35] found significant negative correlations between TIMP-1 immunoreactivity and depth of invasion and lymph node metastasis, though a study conducted by Mroczko et al. [36] reported that expression of TIMP-1 in cancer tissue correlated positively with the depth of tumour invasion and nodal metastasis. We did not find associations between TIMP-1 expression and depth of invasion or lymph node metastasis, which was different from other studies. The inconsistent results may be due to the characteristics of GC patients in our study being different from those of the other studies. Indeed, more than 95% of our patients received radical gastrectomy and had T3- or T4-stage disease, which may have limited the statistical power for evaluating associations between the two proteinases and the tumour invasion process.

Although our results suggested that expression of MMP-7 or TIMP-1 alone cannot serve as an indicator for patient prognosis, there was a significant association between coexpression of MMP-7 and TIMP-1 with poor survival of patients with gastric carcinoma. On the one hand, expression of MMP-7 gradually increases with tumour progression, and eventually, highly expressed MMP-7 induces expression of TIMP-1 to regulate proteinase reactions [37]. The coexpression of MMP-7 and TIMP-1 in tumour tissue reflects a high level of activation of the entire proteinase system, which indicates an increase in tumour growth and metastasis [38]. On the other hand, TIMP-1 mediates constitutive activation of focal adhesion kinase (FAK), and phosphorylated FAK activates phosphatidylinositol 3-kinase (PI3K), which in turn activates Akt. The PI3K/Akt pathway is a canonical pathway involved in antiapoptosis and prosurvival, regulating a number of activities, such as proliferation, migration, and survival [39]. Moreover, the activated PI3K/Akt pathway can further promote expression of MMP-7 downstream [40]. Therefore, we speculate that coexpression of MMP-7 and TIMP-1 in tumour tissue may initiate a positive feedback pathway that directly accelerates tumour invasion and progression. Overall, concomitant positive expression of MMP-7 and TIMP-1 might comprehensively evaluate the state of tumour progression and predict the prognosis of patients with GC.

Our results indicate a positive correlation between concomitant expression of MMP-7 and TIMP-1 and the process of metastasis, which is consistent with the results of our stratified analysis showing that the predictive value was stronger in patients with N3-stage disease (HR: 2.54, 95% CI: 1.21-5.32). This also indicates that for patients with coexpression of MMP-7 and TIMP-1, more attention should be paid to lymph node detection to identify all possible metastatic lymph nodes. Furthermore, the results showed that in patients who did not receive chemotherapy, coexpression of MMP-7 and TIMP-1 could predict poor survival; however, in the subgroup of GC patients who received chemotherapy, the difference disappeared. Previous studies have demonstrated that chemotherapy may have a direct effect on biomarker levels in some patients [41, 42]. TIMP-1 has broad tumour-promoting activity (proangiogenesis, antiapoptosis; promotion of cell growth, and proliferation) [43]. The majority of conventional chemotherapeutic drugs act by killing cancer cells by inducing apoptosis or function as epidermal growth factor receptor inhibitors or antiangiogenic drugs [44, 45]. The effect of apoptosis or antiangiogenesis induced by chemotherapy may affect the functions of TIMP-1 [46–48], which further influences MMP-7 downstream. Therefore, the predictive value of coexpression of MMP-7 and TIMP-1 in the subgroup of patients who received chemotherapy was overshadowed.

Elevation of blood MMP-7 and TIMP-1 levels is considered to result from reactive production of MMP-7 and TIMP-1 in cancerous tissues [49]. Increased blood MMP-7 and TIMP-1 levels occur when the proteins enter the circulation from tumour tissues [50]. Unfortunately, no study to date has investigated the correlation between serum/plasma levels of TIMP-1 and tumour tissue levels of TIMP-1 in GC patients [51]. And only a weak correlation between the serum level of MMP7 and the tumour tissue level of MMP7 has been reported in GC [52]. A study investigating the serum levels of TIMP-1 and MMP-7 in patients with metastatic CRC found that serum MMP-7 together with TIMP-1 can be used as effective tumour markers for detecting metastatic CRC [24]. In GC patients, most studies evaluating blood TIMP-1 levels [53, 54] have reported elevated TIMP-1 protein levels to be associated with a worse prognosis. However, the meta-analysis conducted by Soleyman-Jahi et al. [55] to explore the association between MMP7 and the prognosis of GC indicated that the pooled HR of two studies that used serum MMP7 was not statistically significant. The blood levels of MMP-7 together with TIMP-1 in predicting survival outcome in GC patients have not yet been reported. Noticeably, when using thawed serum samples, there is a substantial risk of falsely elevated levels of TIMP-1 because freezing and thawing of serum have been shown to cause high TIMP-1 levels due to activation and disintegration of platelets, which have a high TIMP-1 content [56]. In addition, serum or plasma levels of MMP-7 are elevated in *Helicobacter pylori* infection and periodontitis [57–59]. Therefore, it should be confirmed whether serum measurements of MMP-7 and TIMP-1 yield reliable results, and further studies are needed to evaluate whether the serum level and tissue expression of the same biomarker correlate in predicting the prognosis of GC.

The tissue microarray method allows analysis of only small regions of each tumour tissue; however, the samples from patients enrolled in a study can be stained as one batch without the risk of heterogeneity between staining experiments. The mechanism of the synergy between MMP-7 and TIMP-1 needs to be further confirmed. Moreover, the clinical application of immunohistochemical staining for MMP-7 and TIMP-1 in combination should be explored on a larger scale to estimate their value as potential predictive biomarkers for therapeutic decision-making and consequently improving survival outcomes.

## 5. Conclusions

In conclusion, coexpression of MMP-7 and its inhibitor TIMP-1 in gastric tumour tissues is a potential prognostic marker for GC. A better understanding of the expression of proteins will lead to new paradigms and possible improvements in therapeutics. Our results are promising but need to be validated in other patient cohorts.

## Figures and Tables

**Table 1:** Clinicopathological characteristics of 285 patients and immunohistochemical staining for MMP-7 and TIMP-1.

Variables		*N* (%)	MMP-7		TIMP-1	
			+ (%)	*P*	+ (%)	*P*
Sex				0.02		0.06
	Male	219 (76.8)	83 (37.9)		54 (24.7)	
	Female	66 (23.2)	15 (22.7)		24 (36.4)	

Age				0.55		0.37
	<70	212 (74.4)	75 (35.4)		61 (28.8)	
	≥70	73 (25.6)	23 (31.5)		17 (23.3)	

T stage				0.71		0.67
	T1	4 (1.4)	1 (25.0)		1 (25.0)	
	T2	10 (3.5)	2 (20.0)		1 (10.0)	
	T3	148 (51.9)	49 (33.1)		40 (27.0)	
	T4	123 (43.2)	46 (37.4)		36 (29.3)	

N stage				0.03		0.67
	N0	47 (16.5)	8 (17.0)		11 (23.4)	
	N1	48 (16.8)	16 (33.3)		13 (27.1)	
	N2	77 (27)	33 (42.9)		25 (32.5)	
	N3	113 (39.6)	41 (36.3)		29 (25.7)	

Differentiation				0.74		0.48
	Middle	58 (20.4)	21 (36.2)		18 (31.0)	
	Low	227 (79.6)	77 (33.9)		60 (26.4)	

Type				0.59		0.73
	Adenocarcinoma and others	255 (89.5)	89 (34.9)		69 (27.4)	
	Signet-ring cell	30 (10.5)	9 (30.0)		9 (30.0)	

Size				0.30		0.61
	<5 cm	85 (29.8)	33 (38.8)		25 (29.4)	
	≥5 cm	200 (70.2)	65 (32.5)		53 (26.5)	

Lymph vascular invasion				0.76		0.63
	No	64 (22.5)	21 (32.8)		16 (25.0)	
	Yes	221 (77.5)	77 (34.8)		62 (28.1)	

Neural invasion				0.002		0.11
	No	78 (27.4)	16 (20.5)		16 (20.5)	
	Yes	207 (72.6)	82 (39.6)		62 (30.0)	

Chemotherapy				0.13		0.07
	No	160 (56.1)	61 (38.1)		37 (23.1)	
	Yes	125 (43.9)	37 (29.6)		41 (32.8)	

+: positive expression.

**Table 2:** Association between the clinicopathological characteristics and prognosis of patients with gastric carcinoma.

Variables		5-year survival (%)	MST (months)	Log-rank *P*
Sex				0.54
	Male	41.5	35.2	
	Female	37.3	26.2	

Age				<0.01
	<70	45.4	42.2	
	≥70	26.9	24.3	

T stage				<0.01
	T1	50.0	50.3^†^	
	T2	77.1	48.8^†^	
	T3	52.3	69.3	
	T4	23.9	22.3	

N stage				<0.01
	N0	71.9	75.2^†^	
	N1	58.2	72.2	
	N2	38.6	27.3	
	N3	20.2	17.1	

Differentiation				<0.01
	Middle	58.6	72.2	
	Low	35.9	26.9	

Type				0.25
	Adenocarcinoma and others	42.3	31.4	
	Signet-ring cell	25.6	28.2	

Size				0.08
	<5 cm	45.4	42.6	
	≥5 cm	38.6	26.9	

Lymph vascular invasion				<0.01
	No	57.0	72.18	
	Yes	35.8	26.94	

Neural invasion				<0.01
	No	64.3	83.15	
	Yes	31.8	24.48	

Chemotherapy				<0.01
	No	32.4	22.28	
	Yes	51.1	83.15	

MMP-7				0.39
	—	41.9	35.58	
	+	37.8	24.81	

TIMP-1				0.71
	—	41.5	35.15	
	+	38.1	27.27	

TIMP-1&MMP-7				0.18
	Both −	41.7	30.82	
	−/+ or +/−	41.9	42.09	
	Both +	30.0	17.38	

TIMP-1&MMP-7				0.07
	Not both +	41.7	37.19	
	Both +	30.0	17.38	

^†^Mean survival time was provided when MST could not be calculated; MST: median survival time, months; −: negative expression; +: positive expression; both −: patients whose tumour tissue expressed neither MMP-7 nor TIMP-1; −/+: patients whose tumour tissue expressed only TIMP-1; +/−: patients whose tumour tissue expressed only MMP-7; both +: patients whose tumour tissue expressed both MMP-7 and TIMP-1.

**Table 3:** Stepwise Cox regression analysis of the survival of gastric cancer patients.

Variables	Adjusted HR (95% CI)	*P*
Age (≥70 vs <70)	1.56 (1.11-2.20)	0.01
T stage	1.39 (1.03-1.88)	0.03
N stage	1.63 (1.35-1.97)	<0.01
Chemotherapy	0.56 (0.40-0.78)	0.01
TIMP-1&MMP-7 (both + vs not both +)	1.74(1.08-2.80)	0.02

Age group, sex, tumour size, T stage, N stage, differentiation, histological type, chemotherapy, lymph vascular invasion, neural invasion, and expression of MMP-7 and TIMP-1 were used as variables in the regression model. Both +: patients whose tumour tissue expressed both MMP-7 and TIMP-1.

## Data Availability

The data used to support the findings of this study are available from the corresponding author upon request.
